# Correlates of climate change skepticism

**DOI:** 10.3389/fpsyg.2024.1328307

**Published:** 2024-04-24

**Authors:** Jona Leka, Adrian Furnham

**Affiliations:** ^1^Department of Experimental Psychology, Division of Psychology and Language Sciences, University College London, London, United Kingdom; ^2^Department of Leadership and Organisational Behaviour, Norwegian Business School (BI), Oslo, Norway

**Keywords:** climate change, skepticism, climate change denial, Just World Beliefs, political beliefs

## Abstract

While much research has examined the correlates of climate change beliefs from an alarmist perspective, less work has systematically measured climate change skepticism. This study aims to create a comprehensive tool capturing climate skeptics’ beliefs and test its association with individual difference variables. 502 European adults completed a 22-item questionnaire on climate change (CC) skepticism as well as measures of ambiguity tolerance, belief in a just world (BJW), dark-side personality traits, and self-esteem. Principal components analysis revealed a four dimension structure of CC. Political ideology was the most consistent and significant predictor across the climate change skepticism factors. Dark-side traits, also played a role. Future research should further validate this measure and explore how climate change information could be tailored to different audiences. Understanding the nuances and causes of climate skepticism can enable more effective communication to promote sustainability.

## Introduction

Anthropogenic climate change is a momentous challenge, with grave consequences for human societies and the natural ecosystems. Despite overwhelming consensus from the scientific community and frequent alerts as to the dangers of current fossil-fuel based practices, a large number of people continue to resist policies intended to curb carbon emissions. A section of the public is unwilling to incur the costs of changing energy sources. However, the most alarming are those who oppose solutions to climate change because they do not believe the assertions made by scientists that anthropogenic climate change is real (Bolsen et al., 2015; Douglas and Sutton, 2015; Carmichael and Brulle, 2017).

As a result, climate change remains a highly divisive issue. This has led to various labels attached to people like “alarmists” and “deniers”: the former insisting on immediate and dramatic changes around a wide range of behaviors, while the latter deny many of the central claims of the climate change scientists. One central question of interest is what personal characteristics are associated with these two extreme groups. Also, nearly all studies concerning beliefs about climate change use statements and concepts from an “alarmist” position while this study does the opposite using primarily “denial” concepts.

There are also other less extreme groups that could be labeled “skeptics” who challenge some of the scientific reports and recommendations and the “concerned” who are worried by, and eager to reverse, some aspects of climate change. Whether a government chooses to initiate mitigation and adaptation policy efforts highly depends on public beliefs and perception of climate change risks. Consequently, research efforts have primarily concentrated on the deniers who, it is suggested, are essentially conspiracy theorists (Hornsey et al., 2018). Strong oppositions from this portion of the public have thwarted efforts to create a low-carbon economy and have caused controversy for a number a renewable energy development (Devine-Wright et al., 2022).

Inevitably, the public debate on climate change has attracted researchers from many different disciplines (Leiserowitz, 2006; Egan and Mullin, 2012; Goeminne, 2012; Leiserowitz et al., 2013; Bolsen et al., 2015; van der Linden, 2015; Dunlap et al., 2016; Pepermans and Maeseele, 2016; Carmichael and Brulle, 2017; Lahn and Sundqvist, 2017; Saunders, 2017; Uscinski and Olivella, 2017; Sellbom et al., 2020; Zummo et al., 2020; Tangney, 2021). This literature seems to have been dominated by philosophers, political scientists and sociologists who inevitably focus on the issue through their particular theoretical and research lenses. Although there is a great deal of work by psychologists on conspiracy theories and inoculation from climate change disinformation (Uscinski and Olivella, 2017; Cook et al., 2018; Compton et al., 2021), less work has been dedicated to measuring climate change skepticism in a systematic way. This is an important gap as by some accounts’ skeptic beliefs may represent a softer side of the denialist claims (Whitmarsh, 2011). Understanding these attitudes in turn may inform campaigns aimed at increasing compliance with mitigation policies (Spence and Pidgeon, 2009). Thus, the aim of the current study is to create a comprehensive tool that encapsulates climate skeptics beliefs. More importantly relatively few studies have looked at individual difference variables like personality or belief systems. In this study we explore both, such as the Just World Belief construct which is concerned with concepts of fate and deservingness. Further we examine “dark-side” personality traits which have been linked to beliefs in conspiracy theories (Furnham and Grover, 2021). We also examine self-esteem and whether this is related to climate change beliefs.

Before delving into the details of the study, a general description of climate change denial is warranted. There are five techniques associated with denialists arguments: conspiracy theories, fake experts, impossible expectations, misrepresentations and logical fallacies, and cherry- picking (Diethelm and McKee, 2009). These techniques help people maintain their doubts about the occurrence of climate change, its causes and impacts. Where one falls on the climate change skepticism spectrum then depends on the form of denial employed. This in turn reflects the problematic way in which the public perceives climate change information. Poortinga et al. (2011) notes that this has created enormous heterogeneity in the construct. Rahmstorf (2004) distinguishes between *trend skeptics* who deny climate change; *attribution skeptics*, who do not deny that the climate may be changing, but deny human involvement; and *impact skeptics* who do not deny human-caused climate change, but do not believe it will have detrimental impacts. Leiserowitz et al. (2021) extended this categorization in the US to comprise six audience segments based on their responses to climate change information, which they have termed the six Americas: *The Alarmed, Concerned, Cautious, Disengaged, Doubtful, and Dismissive.* In 2020 the latter three made up 26% of the US population and typically represent climate deniers in line with Rahmstorf’s (2004) categorization. However, recent findings have shown climate change to be a more politically fueled issue in the US than in other countries, which renders this categorization difficult to apply to other populations (Hornsey et al., 2018).

In recent years, the terms ‘deniers’, ‘contrarians’ ‘dissenters’ and ‘skeptics’ have been used interchangeably to describe the portion of the public that take scientific uncertainty as an absence of consensus on climate change, or who publicly misrepresent climate science and attack scientific claims (McCright, 2007; Anderegg and Harold, 2009). This is in line with Tobler et al. (2012) and Malpass et al. (2007) who argue that climate skeptics tend to distrust scientific facts and harbor doubts about individual responsibility for climate change. However, Whitmarsh (2011) noted that climate change denial is a multidimensional construct comprised of both an active rejection of climate science, and more general attitudinal ambivalence and uncertainty about climate change. The portion of the public who is uncertain about the facts of climate change is typically larger (Leiserowitz et al., 2010, 2021). Having a multitude of constructs under pejorative terms such as ‘climate dissenter’ has led to a lack of clarity regarding the proportion of the public that endorses such claims. Moreover it hampers attempts to establish a synergistic relationship between climate science, policy and society (O’Neill and Boykoff, 2010).

Therefore, we sought to construct a comprehensive tool that reliably measures climate change deniers beliefs, as well as test its association to individual constructs that typically predict skeptical beliefs.

### Psychological correlates of climate denialism

Psychological research has shown that adopting denialist thinking is linked to absence of control (Whitson and Galinsky, 2008), uncertainty (Whitson et al., 2015) and perceived self-esteem threats (Cichocka et al., 2015). This is especially true in the case of existential threats such as climate change, which put the desire for control in jeopardy and frame unsustainable actions as malevolent (Uscinski and Olivella, 2017; Douglas et al., 2019) thus potentially threatening one’s identity. These reactions are further complicated by political ideologies, as seen in the relationship between support for Trump’s policies, aversion to wealth redistribution, and climate change skepticism, especially among those with high political interest (Panno et al., 2019). Moreover, conservative political orientation, amplified by high political interest, has been shown to predict climate change denial through ideological pathways like right-wing authoritarianism and social dominance orientation (Carrus and Leone, 2018). This suggests that identity-protecting cognition and neoliberal beliefs might interact, often leading to a lowering of risk perceptions and denial of climate change (Haltinner and Sarathchandra, 2018). Thus, public beliefs about climate change are shaped by a complex interplay of individual differences, psychological orientations, political ideologies, and knowledge of climate science (Whitmarsh, 2011). A question of interest then is how people’s identities as well as their way of coping with the inherent uncertainty of climate change impact climate change beliefs. To explore this, we included measures of Tolerance of Ambiguity and Self-esteem to explore their relation to climate change skepticism.

Another question concerns how diverse worldviews shape the interpretation of climate change information (Hornsey, 2021). A worldview that is particularly relevant to climate change skepticism is Belief in a Just World: The BJW concept was identified over 50 years ago and concentrates on the tendency of people to blame victims of misfortunes for their own fate (Lerner and Miller, 1978; Lerner, 1980). The central idea is that people have a fundamental need to believe that the (social) world is a just place and that this belief is functionally necessary for them to develop principles of deservingness. People are confronted with difficult social problems such as why some people get ill, are abused, descend into poverty etc. while others do not. They also have to make sense of their own misfortunes. The idea of the BJW is that it helps answer some of these very difficult moral, political and social questions. Previous studies have shown that people holding BJW tend to be more skeptical of climate change, especially when messages are expressed in terms of the detrimental consequences of climate change for human societies (Feinberg and Willer, 2011). It is thought that such messages contradict the belief that the world is just, thus producing denial. Therefore, we were interested in exploring how this factor relates to climate change beliefs.

Finally, we were interested in examining the relationship between personality and climate skepticism. Many studies have tested the association between Personality measures and climate change beliefs. Several studies have shown that Big-5 traits such as Openness and Conscientiousness favorably influence climate beliefs and sustainable behavior (Brick and Lewis, 2016). Here we were interested in the relatively underexplored dark-side personality measures (essentially sub-clinical personality disorders) and climate skepticism. These have been as measured by the new five-dimensional measure (PID-5-BF). This has five dimensions:

I. *Negative Affectivity* (Anxiousness, Emotional lability, Hostility, Perseveration, Lack of restricted affectivity, Separation insecurity, Submissiveness), II. *Detachment* (Anhedonia, Depressivity, Intimacy avoidance, Suspiciousness, Withdrawal), III. *Antagonism* (Attention seeking, Callousness, Deceitfulness, Grandiosity, Manipulativeness), IV. *Disinhibition* (Distractibility, Impulsivity, Irresponsibility, Lack of rigid perfectionism, Risk taking), and V*. Psychoticism* (Eccentricity, Perceptual dysregulation, Unusual beliefs and experiences). Several studies have shown that dark personality traits such as Antagonism are typically associated with need for control, insensitivity to untrustworthiness cues, and gullibility which renders people vulnerable to conspiracy beliefs (Hart et al., 2021; Miller et al., 2021; Cichocka et al., 2022). Considering the vast literature on the link between climate change denial and conspiratorial ideation we sought to explore whether such traits could account for climate change skeptics’ beliefs.

### Aims of the study

The first innovative aspect of the study is our measure of CC beliefs. While there are a number of measures in this area, there is no ‘gold standard’ for measuring climate change beliefs with psychometrically robust and proven tools. Various studies have employed a multiple-choice format in which respondents are asked to select the factors they believe cause climate change. A variation of this is asking people to indicate the degree to which they believe climate change is caused by human activities (Poortinga et al., 2011; Goldberg et al., 2019). However, most researchers construct their own measurement tools and infer degree of belief in climate change from the level of agreement with various statements. These measures tend to be characterized by two factors. The first, is that tend to be short and often of unproven psychometric validity.

The second is that they often unashamedly have more items from the alarmist perspective. This in turn has resulted in conflicting reports of climate change beliefs and sometimes artificially low levels of denial. For instance, in 2010 beliefs in anthropogenic climate change ranged from 50.4 to 94% for people in Australia depending on the measurement tool used (Leviston et al., 2011). It is known that subtle wording and response options can have a major impact on test results, thus rendering conclusions drawn from these studies often elusive (Greenhill et al., 2014). One issue of considerable interest to questionnaire researchers is whether agreeing with a positive statement about any issue is much the same as disagreeing with a negative statement. Therefore, in this study we sought to craft a questionnaire from the denialist perspective in order to assess its validity in measuring climate change skepticism.

The first phase of the study involved the selection of several statements that are in line with the climate change denial. There have been a wealth of papers from many disciplines on the beliefs of deniers which are very clearly documented and set out in a comprehensive entry into Wikipedia. This was a source of climate skeptics’ statements.

The first aim of the study was to test the reliability of the CC instrument in measuring climate skeptics beliefs. The second aim was to test how these beliefs associate with various attitudinal, demographic, personality and worldview measures. We expected based on previous research that BJW would be positively associated with CC skepticism (H1). Based on the literature on identity protecting cognition we also expected that TOA would be negatively associated with CC skepticism (H2) and Self-esteem would be negatively associated with CC Skepticism (H3). As there is no extensive research on the dark side personality measures and CC skepticism we made a tentative prediction that dark side DSM factors would be positively associated with CC skepticism (H4) based on the general characteristics associated with each dimension of personality.

## Method

### Participants

There were 502 participants, 254 males and 248 females. They ranged in age from 30–69 years old, with a modal age of 36. In all 70.9% were graduates. With regard to their religious beliefs (1 = *Not At All* to 9 = *Very*), they scored a mean of 3.80 (*SD* = 3.01). They rated their political views from (1) Very Conservative to (9) Very Liberal, with a mean of 5.83 (*SD* = 1.81). They rated “I am an optimist” from (10) Agree to (1) Disagree, with a mean of 6.74 (*SD* = 2.15). Asked 2% said they were vegan, 7% vegetarian and the remainder (91%) neither. They stated their religious views on (1) Not religious to (9) Very Religious scale with a mean score of 2.88 (SD = 2.54).

### Questionnaires

#### Measures

##### Climate change

This questionnaire was created for this study and based on many statements from the *Wikipedia* entry on Climate Change Denial. Twenty-two statements were mainly taken from this entry as well as other papers on the topic. These are shown in Table 1. They were piloted for clarity. The instructions read *“There is still a lot of debate about climate change. This short questionnaire asks you to rate how much you agree or disagree about a number of statements that* var*ious people have made about the topic**.”***

**Table 1:** Means and SDs for each of the 22 items (9 = very strongly agree; 1 = very strongly disagree).

Item	Mean	SD
1. The science behind global warming has been invented or distorted for ideological or financial reasons.	2.53	2.02
2. It is extremely likely (95–100%) that human influence has been the dominant cause of the observed warming since the mid-20th century.	7.05	1.90
3. Hundreds of climate scientists have twisted their results to support the climate change theory in order to protect their research funding.	2.72	1.97
4. The climate has, and will, always change dramatically.	5.76	1.98
5. Nearly all scientists who have studied climate change are in total agreement.	5.63	2.20
6. Researchers faked the data in their research publications and suppressed their critics in order to receive more funding (i.e., taxpayer money).	2.44	1.72
7. Peer-review process for papers in climate science has become corrupted by scientists seeking to suppress dissent.	2.99	1.90
8. It was easy for climate scientists to manipulate the data to come up with the results they want to make headlines and drive their environmental agendas.	3.11	2.04
9. With all of the hysteria, fear, and phony science, the idea of man-made global warming is the greatest hoax ever perpetrated on the European countries.	2.36	1.93
10. We, humans, are primarily responsible for climate change.	7.13	1.86
11. Global warming is a multi-billion-dollar worldwide industry, created by fanatically anti-industrial environmentalists.	2.80	2.15
12. Climate science is less about science and more about socialist ideology.	2.86	2.09
13. Environmental groups bribe climate scientists to doctor their data so that they are able to secure their financial investment in green energy.	2.32	1.74
14. The concept of global warming was created by, and for, the Chinese in order to make western manufacturing non-competitive.	1.89	1.39
15. The threat of global warming is an attempt to promote nuclear power.	2.18	1.54
16. Global warming is causing the migration routes of many birds, fish, mammals, and insects to change.	6.78	1.92
17. Climate conspiracies are part of well-funded misinformation campaigns designed to manufacture controversy, and undermine the scientific consensus on climate change.	3.71	2.44
18. A well-coordinated, well-funded campaign by contrarian scientists, free-market think tanks and industry has created a paralyzing fog of doubt around climate change.	3.92	2.19
19. ExxonMobil distributed US$2.9 million to 39 groups that misrepresented the science of climate change by outright denial of the evidence.	4.30	1.78
20. There are as many benefits as drawbacks from climate change.	2.93	1.85
21. It is pointless for America and Europe to change their lifestyle when China and India continue to build coal-fired power stations.	3.74	2.53
22. Global warming is a punishment from God for the way we have treated his earth.	1.78	1.67

##### Self-esteem

The self-esteem measure (Furnham et al., 2020) comprised four other factors on a scale from 1–100: Physical Attractiveness (*M* = 52.58; *SD* = 17.58), Physical Health (*M* = 61.76, *SD* = 19.93), Intelligence (IQ) (*M* = 67.88, *SD* = 14.28) and Emotional Intelligence (*M* = 67.35, *SD* = 16.68).

The Alpha for these four items was 0.69, and they were summed together forming a variable labeled Self-Esteem.

##### Global Belief in a Just World

This is a seven-item measure. Hellman et al. (2008) found the Global Belief in a Just World Scale (Lipkus, 1991) produced the highest average reliability score (*α* = 0.81) compared to the Just World Scale (Rubin and Peplau, 1973; *α* = 0.64) and the Just World Scale Revised (Rubin and Peplau, 1975; *α* = 0.68). In this study the alpha was 0.90.

##### Tolerance of ambiguity

This is a 12 item scale by Herman et al. (2010) which was devised to improve on the earlier scale of Budner (1962) with an alpha of 0.73. The authors demonstrate an improved factor structure and internal consistency for the TOA compared to the measure of Budner.

##### DSM-5—brief form (PID-5-BF)

The Personality Inventory for DSM (Krueger et al., 2012; Díaz-Batanero et al., 2019) is a 25-item self-rated assessment scale which assesses five personality trait domains [Negative Affect (0.74), Detachment (0.60), Antagonism (0.68), Disinhibition (0.72) and Psychoticism (0.75)] with each trait domain consisting of 5 items. It has been validated by a number of psychometric studies in different countries (Al-Dajani et al., 2016; Sellbom et al., 2020).

### Procedure

Departmental ethical approval was gained prior to data collection. Data was collected on Prolific and participants were compensated for their time at the set rate. Data cleansing took part before the formal analysis.

## Results

Table 1 shows the means and SDs for each of the statements. Scores, on the nine-point scale ranged from 1.78 (Global warming is a punishment from God for the way we have treated his earth) to 7.13 (We, humans, are primarily responsible for climate change). Standard Deviations varied from 1.39 (The concept of global warming was created by, and for, the Chinese in order to make western manufacturing non-competitive) to 2.53 (It is pointless for America and Europe to change their lifestyle when China and India continue to build coal-fired power stations.). In general, the level of CC denial was low-to-moderate. The highest mean agreement score was with the statements aligned with anthropogenic climate change views. This is in line with a general trend in recent years showing level of skepticism to be on the decline (Leiserowitz et al., 2021) and climate change to be considered a serious problem by one in five Europeans. It is noteworthy that this trend appears to hold independently of whether questions are framed from the denialist perspective or the alarmist perspective.

These data were then treated to a Varimax and Promax rotated principal component factor analysis. The results of both analyses were very similar. We used results from the Varimax analysis which showed a four factor solution which in total accounted for around ¾ of the variance (see Table 2). The emergent factors have some conceptual similarity with Rahmstorf’s (2004) categorization. The alphas for the four factors were 0.77, 0.70, 0.71 and 0.45, respectively, suggesting the first three were internally reliable and coherent. In particular Factor 1 resembles both *trend skepticism and attribution skepticism* whereas Factor 4 is conceptually similar to *impact skepticism.* In general, these represent the uncertainty relating to the veracity of Climate science claims, thus forming the basis of the CC skepticism scale. It is noteworthy that items corresponding to climate conspiracies and misinformation loaded more strongly on Factor 3 suggesting these might be conceptually different from general misinformation and climate science skepticism.

**Table 2:** PCA analysis of the 22 items.

	1	2	3	4
CCQ 10 We, humans, are primarily responsible for climate change	**−0.825**	0.153	0.058	−0.118
CCQ 2 It is extremely likely (95–100%) that human influence has been the dominant cause of the observed warming since the mid-20th century	**−0.807**	0.120	0.028	−0.112
CCQ 1 The science behind global warming has been invented or distorted for ideological or financial reasons.	**0.780**	0.265	0.080	0.238
CCQ 12 Climate science is less about science and more about socialist ideology.	**0.780**	0.275	0.043	0.307
CCQ 7 Peer-review process for papers in climate science has become corrupted by scientists seeking to suppress dissent.	**0.770**	0.256	0.116	0.295
CCQ 8 It was easy for climate scientists to manipulate the data to come up with the results they want to make headlines and drive their environmental agendas.	**0.753**	0.295	0.078	0.337
CCQ 3 Hundreds of climate scientists have twisted their results to support the climate change theory in order to protect their research funding	**0.746**	0.347	0.054	0.277
CCQ 6 Researchers faked the data in their research publications and suppressed their critics in order to receive more funding (i.e., taxpayer money)	**0.741**	0.325	0.092	0.243
CCQ 11 Global warming is a multi-billion-dollar worldwide industry, created by fanatically anti-industrial environmentalists.	**0.733**	0.328	0.126	0.314
CCQ 9 With all of the hysteria, fear, and phony science, the idea of man-made global warming is the greatest hoax ever perpetrated on the European countries.	**0.732**	0.357	0.073	0.241
CCQ 13 Environmental groups bribe climate scientists to doctor their data so that they are able to secure their financial investment in green energy.	**0.682**	0.491	0.157	0.144
CCQ 16 Global warming is causing the migration routes of many birds, fish, mammals, and insects to change.	**−0.672**	0.084	0.258	0.104
CCQ 5 Nearly all scientists who have studied climate change are in total agreement.	**−0.428**	0.041	0.317	−0.164
CCQ 22 Global warming is a punishment from God for the way we have treated his earth.	−0.083	**0.785**	−0.022	0.131
CCQ 15 The threat of global warming is an attempt to promote nuclear power.	0.507	**0.618**	0.131	0.074
CCQ 14 The concept of global warming was created by, and for, the Chinese in order to make western manufacturing non-competitive.	0.566	**0.612**	0.161	0.016
CCQ 18 A well-coordinated, well-funded campaign by contrarian scientists, free-market think tanks and industry has created a paralyzing fog of doubt around climate change	0.085	0.093	**0.857**	0.019
CCQ 17 Climate conspiracies are part of well-funded misinformation campaigns designed to manufacture controversy, and undermine the scientific consensus on climate change.	0.046	−0.015	**0.755**	0.168
CCQ 19 ExxonMobil distributed US$2.9 million to 39 groups that misrepresented the science of climate change by outright denial of the evidence.	−0.025	0.051	**0.728**	−0.100
CCQ 4 The climate has, and will, always change dramatically.	0.160	0.006	−0.047	**0.859**
CCQ 20 There are as many benefits as drawbacks from climate change	0.426	0.270	0.042	**0.543**
CCQ 21 It is pointless for America and Europe to change their lifestyle when China and India continue to build coal-fired power stations.	0.431	0.184	0.086	**0.531**
Eigenvalue	10.170	2.246	1.273	1.073
Variance	46.227%	10.211%	5.787%	4.875%

Rotation is Varimax with Kaiser normalization.

The overall scale showed good internal consistency with a Cronbach *α* = 0.83. There were no problems concerning sampling adequacy or sphericity based on KMO statistics (0.94) and Bartlett’s test (*p* < 0.001). The rest of the scales: Self-esteem, Belief in a Just World, Tolerance of Ambiguity, and DSM factors were collapsed to form a singular score.

### Correlational analysis

We performed correlational analysis for all variables of interest and the four factors of CC skepticism (see Table 3). The highest correlations were obtained between BJW and CC one (*r* = 0.17) and CC four (*r* = 0.21), TOA and CC two (−0.16) and CC four (−0.12). In terms of Personality, positive correlations emerged between Detachment and CCthree, Disinhibition and Psychotism and CC two (*r* = 0.18 and *r* = 0.16 respectively) and CCthree (*r* = 0.16 and *r* = 0.15 respectively), and Self- Esteem and CCthree (*r* = −11). Table 3 shows some of the hypotheses were confirmed for certain factors of CC skepticism. The expected negative correlation between TOA, Self-esteem and CC skepticism is in line with previous studies (Jessani and Harris, 2018) and predictions. And the predicted positive association between BJW and CC skepticism was obtained for three out of the four factors in line with previous findings (Feinberg and Willer, 2011). Positive associations were obtained between dark side personality traits and all CC skepticism factors in line with predictions.

**Table 3:** Correlation between CC factors and scales.

	Mean	SD	1	2	3	4	5	6	7	8	9	10	11
1. CCone	25.8	14.8											
2. CCtwo	5.8	3.6	0.641^***^										
3. CCthree	11.9	5.1	0.118^*^	0.206^***^									
4. CCfour	12.4	4.9	0.658^***^	0.448^***^	0.108*								
5. SELF	249.2	49.3	−0.084	−0.101^*^	−0.110^*^	−0.086							
6. BJW	29	9.9	0.170^***^	0.156^**^	−0.115^*^	0.206^***^	0.168^***^						
7. TOA	63	10.4	−0.053	−0.158^**^	−0.057	−0.122^*^	0.118^*^	−0.103^*^					
8. DSMNegAff	5.2	3.3	0.013	0.190^***^	0.108^*^	0.070	−0.281^***^	−0.116^*^	−0.280^***^				
9. DSMDetach	4.1	3.1	0.046	0.088	0.206^***^	0.140^**^	−0.381^***^	−0.053	−0.243^***^	0.467^***^			
10. DSMAntag	2.1	2.2	0.083	0.096	0.157^**^	0.081	−0.002	0.169^***^	−0.059	0.192^***^	0.308^***^		
11. DSMDisinhib	2.5	2.7	0.096	0.180^***^	0.162^**^	0.136^**^	−0.197^***^	−0.018	0.041	0.274^***^	0.307^***^	0.424^***^	
12. DSMPsychot	3.3	3.0	0.105^*^	0.165^***^	0.152^**^	0.127^*^	−0.249^***^	−0.074	−0.079	0.496^***^	0.493^***^	0.440^***^	0.552^***^

*p < 0.05, **p < 0.01, ***p < 0.001.

We were also interested in exploring the relationship between political ideology and climate change skepticism. We found a strong negative correlation between political ideology and most CC skepticism factors, such that the more right leaning tended to be more skeptical of climate change. This is a well-documented finding in the literature (Hornsey et al., 2016). However, a significant positive relationship emerged between political ideology and CCthree (*r* = 0.13) and BJW and CC three (*r* = 0.12) contrary to predictions, which might be because this factor is measuring a unique dimension of CC skepticism.

### Regression analysis

Linear regressions were performed between demographic variables, education, political ideology, BJW, Self-esteem, TOA, DSM and the CC skepticism factors. These are shown in Table 4. The regression analyses revealed that DSM Disinhibition emerged as a significant predictor for CC Two and CC Four. Political orientation was found to be a notable predictor for the majority of the CC factors. Education level was a significant predictor for three out of the four factors. Religious beliefs were found to significantly influence CC Two. Age and gender also contributed to the prediction model, with age emerging as a significant predictor for the first CC factor and gender for the third. The whole model tests were significant with reasonable fit accounting for a good proportion of variance in skepticism R^2^ = 0.16 for CCOne, R^2^ = 0.16 for CCTwo, R^2^ = 0.11 for CCThree, and R^2^ = 0.19 for CCFour.

**Table 4:** Regression onto the four factors.

	CC One				CC Two				CC Three				CC Four			
	std. Beta	*SE*	*t*	*p*	std. Beta	*SE*	*t*	*p*	std. Beta	*SE*	*t*	*p*	std. Beta	*SE*	*t*	*p*
Sex	−0.01	1.54	−0.22	0.824	0.04	0.38	0.78	0.434	−0.20	0.55	−3.79***	<0.001	0.02	0.50	0.32	0.747
Age	−0.11	0.07	−2.23*	0.026	−0.07	0.02	−1.38	0.169	0.01	0.03	0.24	0.809	0.04	0.02	0.85	0.397
Degree	0.11	1.51	2.15*	0.032	0.11	0.37	2.25*	0.025	−0.13	0.54	−2.52*	0.012	0.03	0.50	0.62	0.533
Religious	0.10	0.30	1.98	0.049	0.15	0.07	3.05**	0.002	−0.05	0.10	−1.04	0.300	0.04	0.10	0.74	0.460
Political	−0.35	0.40	−6.57***	<0.001	−0.21	0.10	−3.94***	<0.001	0.08	0.14	1.45	0.149	−0.37	0.13	−7.01***	<0.001
BJWtot	0.02	0.08	0.30	0.762	0.07	0.02	1.26	0.208	−0.10	0.03	−1.80	0.073	0.06	0.03	1.06	0.291
SELF	−0.04	0.20	−0.67	0.505	−0.03	0.00	−0.52	0.601	−0.06	0.01	−1.05	0.292	0.01	0.01	0.10	0.922
ToaTot	−0.02	0.07	−0.30	0.766	−0.09	0.02	−1.81	0.072	−0.04	0.03	−0.69	0.489	−0.08	0.02	−1.69	0.091
DSMNegAff	−0.06	0.28	−0.93	0.353	0.10	0.07	1.69	0.091	0.06	0.10	1.02	0.309	−0.01	0.09	−0.18	0.854
DSMDetach	0.00	0.28	0.06	0.956	−0.03	0.07	−0.49	0.625	0.09	0.10	1.46	0.146	0.08	0.10	1.26	0.207
DSMAntag	0.03	0.38	0.55	0.582	0.01	0.10	0.09	0.927	0.04	0.14	0.76	0.448	−0.06	0.12	−1.00	0.317
DSMDisinhib	0.06	0.33	0.96	0.339	0.16	0.10	2.76**	0.006	0.08	0.02	1.33	0.185	0.15	0.11	2.65**	0.009
DSMPsychot	0.07	0.33	1.12	0.261	0.02	0.10	0.30	0.766	−0.01	0.02	−0.20	0.843	0.04	0.11	0.65	0.514
F	6.943**				6.930***				4.855***				8.216***			
R^2^/R^2^ adjusted	0.192/0.164				0.191/0.164				0.142/0.113				0.219/0.192			

*p < 0.05, **p < 0.01, ***p < 0.001.

In order to examine the relative importance of Personality factors above demographic variables, a hierarchical regression was performed as well onto all CC Skepticism factors. This is shown in Table 5. For CCone, the first model including Age, Religious views, Sex and Political orientation was significant *F*(4,396) = 19.027, *p* < 0.001. This model accounts for R^2^ = 0.16 of the variance in CC one. Looking at the individual predictors, Political views b = −0.39, 95% CI[−3.6,-2.2], *t*(396) = 8.08, *p* < 0.001 have an impact on CC One. The next two models are also significant but none of the added individual predictors reach significance.

**Table 5:** Hierarchical regression onto the 4 factors.

Predictors	CC one												CC two											
	(1)				(2)				(3)				(1)				(2)				(3)			
	Std. Beta	SE	*t*	*p*	Std. Beta	SE	*t*	*p*	Std. Beta	SE	*t*	*p*	Std. Beta	SE	*t*	*p*	Std. Beta	SE	*t*	*P*	Std. Beta	SE	*t*	*p*
Sex	−0.04	1.41	−0.74	0.460	−0.03	1.44	−0.60	0.547	0.00	1.54	0.01	0.995	0.03	0.36	0.58	0.563	0.05	0.36	0,94	0.350	0.05	0.38	0.97	0.332
Age	−0.10	0.07	−2.15*	0.032	−0.11	0.07	−2.19*	0.029	−0.10	0.07	−1.96	0.051	−0.08	0.02	−1.56	0.119	−0.07	0.02	−1.45	0.146	−0.05	0.02	−1.03	0.302
Political	−0.39	036	8.08***	<0.001	0.37	0.40	−6.99***	<0.001	−0.37	0.40	−6.97***	<0.001	−0.26	0.09	5.15***	<0.001	−0.21	0.10	−3.89***	<0.001	−0.23	0.09	−4.30***	<0.001
Religious	0.07	0.28	1.51	0.132	0.08	0.28	1.59	0.114	0.08	0.28	1.55	0.122	0.15	0.07	3.01**	0.003	0.15	0.07	3.00**	0.003	0.13	0.07	2.68**	0.008
BJWtot					0.03	0.08	0.49	0.627	0.02	0.08	0.45	0.655					0.08	0.02	1.44	0.150	0.08	0.02	1.46	0.145
SELF					−0.07	0.01	−1.53	0.128	−0.06	0.02	−1.09	0.276					−0.10	0.00	−2.12*	0.035	−0.05	0.00	−0.94	0.346
ToaTot					−0.01	0.07	−0.19	0.847	−0.02	0.07	−0.48	0.628					−0.11	0.02	−2.26*|	0.025	−0.10	0.02	−2.01*|	0.045
DSMNegAM									−0.05	0.27	−0.82	0.415									0.11	0.07	1.80	0.073
DSMDetach									−0.01	0.28	−0.18	0.854										−0.04 0.07	−0.73	0.469
DSM/Antag									0.02	0.38	0.28	0.776									−0.01	0.09	−0.22	0.822
DSMDisinhib									0.07	0.32	1.16	0.246									0.18	0.08	3.02**	0.003
DSMPsychot									0.08	0.33	1.18	0.238									0.03	0.08	0.40	0.668
F	19.027***				11.226***				7.098***				11.305***					8.452***				7.042***		
R^2^	0.163				0.168				0.182				0.103					0.132				0.180		
R- adjusted	0.154				0.153				0.156				0.094					0.116				0.155		

Predictors	CC three												CC four											
	(1)				(2)				(3)				(1)				(2)				(3)			
	Std. Beta	*SE*	*t*	*p*	Std. Beta	*SE*	*t*	*p*	Std. Beta	*SE*	*t*	*p*	Std. Beta	*SE*	*t*	*p*	Std. Beta	*SE*	*t*	*p*	Std. Beta	*SE*	*t*	*p*
Sex	−0.21	0.51	−4.22***	<0.001	−0.23	0.51	−4.49**	<0.001	−0.21	0.54	−3.91**	<0.001	−0.01	0.47	−0.18	0.857	0.00	0.47	0.07	0.946	0.03	0.49	0.51	0.614
Age	0.00	0.03	0.00	1	−0.01	0.03	−0.18	0.858	−0.01	0.03	−0.26	0.792	0.03	0.02	0.57	0.572	0.03	0.02	0.66	0.509	0.04	0.02	0.92	0.356
Political	0.14	0.13	2.71**	0.007	0.10	0.14	1.86	0.064	0.09	0.14	1.70	0.090	−0.40	0.12	−8.43***	<0.001	−0.37	0.13	−7.11***	<0.001	−0.38	0.13	−7.26***	<0.001
Religious	−0.04	0.10	−0.83	0.408	−0.04	0.10	−0.84	0.402	−0.04	0.10	−0.70*	0.485	0.02	0.09	0.49	0.623	0.02	0.09	0.45	0.650	0.03	0.09	0.60	0.546
BJWtot					−0.11	0.03	1.97*	0.049	−0.12	0.03	−2.10	0.036					0.05	0.02	0.95	0.341	0.06	0.03	1.11	0.268
SELF					−0.09	0.01	−1.83	0.068	−0.03	0.01	−0.64	0.521					−0.07	0.01	−1.43	0.153	−0.01	0.01	−0.12	0.908
ToaTot					−0.06	0.02	−1.21	0.225	−0.02	0.03	−0.48	0.633					−0.09	0.02	−1.82	0.70	−0.08	0.02	−1.66	0.098
DSMNegAM									0.06	0.09	0.90	0.368									−0.01	0.09	−0.13	0.898
DSMDetach									0.11	0.10	1.68	0.094									0.07	0.09	1.21	0.225
DSM/Antag									0.07	0.14	1.18	0.239									−0.06	0.12	−1.03	0.302
DSMDisinhib									0.06	0.12	1.01	0.315									0.15	0.11	2.62**	0.009
DSMPsychot									−0.03	0.12	−0.39	0.697									0.05	0.11	0.69	0.488
F	7.221***				5.792***				4.604***				20.326***					12.734***				8.949***		
R^2^	0.069				0.094				0.126				0.172					0.186				0.219		
R- adjusted	0.059				0.078				0.098				0.163					0.172				0.194		

*p < 0.05, **p < 0.01, ***p < 0.001.

For CC Two the first model was significant *F*(4,397) = 11.305, *p* < 0.001 accounting for R^2^ = 0.09 of the variance. Both Political views b = −0.26, 95% CI[−0.65,-0.29], *t*(397) = 5.15, *p* < 0.001 and Religious views b = 0.15, 95% CI[0.07,0.35], *t*(396) = 3.01, *p* < 0.05 were significant predictors of CC Two. The second model was significant *F*(7,396) = 8.452, *p* < 0.01 and accounts for R^2^ = 0.11 of the variance adding 2% of explained variance. The third model including personality factors was significant *F*(12,396) = 7.04 and adds 4% of explained variance in CCtwo with Disinhibition b = 0.18, 95% CI[0.08,0.39], *t*(397) = 3.02, *p* < 0.01 emerging as a significant predictor of CC Two.

For CC Three the first model is significant *F*(4,396) = 7.221, *p* < 0.001 accounting for R^2^ = 0.06 of the variance. Sex is a significant predictor of CC Three b = −0.21, 95% CI[−3.2,-1.15], *t*(396) = − 4.2, *p* < 0.001. The second model is significant *F*(7,396) = 5.792, *p* < 0.001 and accounts for R^2^ = 0.08 of the variance. Looking at the individual predictors, BJW barely reaches significance. This model adds 2% of explained variance. The third model including personality factors was significant *F*(12,397) = 4.880 and adds 2% of explained variance in CC Three.

For CC Four the first model is significant *F*(4,396) = 4.517, *p* < 0.001 accounting for R^2^ = 0.16 of the variance. Political views is a significant predictor of CC Four b = −0.40, 95% CI[−1.2,-0.77], *t*(397) = −8.43, *p* < 0.001. The second model is significant *F*(7,396) = 12.73, *p* < 0.001 and accounts for R^2^ = 0.17 of the variance. The third model including personality factors was significant *F*(12,396) = 8.94 and adds 2% of explained variance in CC Four. Here Disinhibition emerged as a significant predictor of CC Four b = 0.15, 95% CI[0.07,0.48], *t*(396) = 2.6, *p* < 0.01.

## Discussion

A plethora of research explores the characteristics of environmental attitudes and correlates of climate change beliefs (Hornsey et al., 2016). However, few scholars have attempted to examine climate change beliefs from the denialist perspective. Notable exceptions include work done by Whitmarsh (2011) and Poortinga et al. (2011). The majority of psychometric tools employed to examine Climate Change beliefs tend to include items that may skew responses toward more ‘alarmist’ attitudes. In this study we carried out an investigation of Climate Change beliefs through a new tool constructed from the denialist perspective. The study explored how widespread skeptical beliefs are in Europe and to what extent they are associated worldviews such as Just World Beliefs and Political ideology and attitude aspects, such as Tolerance of ambiguity. We also set out to test the association between Dark Side personality facets and climate change skepticism, a relatively underexplored factor in the climate change attitudes literature.

The majority of participants tended to agree with human-caused climate change and the scientific consensus regarding anthropogenic climate change. These results are in line with a general trend showing certain types of skepticism surrounding climate change may be declining while level of concern is on the rise (Saad, 2017a,b). Other studies have reported alarmed segments to have risen by 9% since 2008 and the Dismissive and Disengaged segments to have dropped by 1 and 9%, respectively, (e.g., Leiserowitz et al., 2021). However, such trend observations should be taken with caution as previous reports mostly concern the US population and use different measurement tools. Most studies have primarily reported on levels of agreement with the causes of climate change as a human-made or naturally occurring phenomenon (Poortinga et al., 2011; Leiserowitz et al., 2021). The current results extend this line of findings by showing that levels of agreement with conspiratorial rhetoric and disinformation surrounding climate change are among the lowest. This suggests that these types of beliefs are not prevalent in the public discourse. Another possibility might be that outright climate change denial might be pervasive only among those who are very right leaning (Roser-Renouf et al., 2014) which made up only 5% of our sample.

The measure of Climate Change beliefs devised from the denialist perspective showed a clear multidimensional structure. Items concerning the trustworthiness of climate scientist claims tended to be quite different from conspiracy-related items and items relating to the impacts of climate change. These observations have been echoed in previous research and deserve further investigation (Poortinga et al., 2011; Whitmarsh, 2011). It might be that a level of uncertainty surrounding climate science is a softer stance than outright denial of scientific data and conspiracy-laden rhetoric and each may be impacted by a different set of attitudinal variables. Exploring these dimensions further may aid intervention attempts aimed at clarifying climate science uncertainties and solidifying the knowledge required to take climate action. Although the instrument devised here showed high reliability, further studies are needed to ascertain its validity in capturing climate skeptics’ beliefs.

Similarly to previous studies (Carrus and Leone, 2018; Panno et al., 2019), we find that certain ideologies tend to be more related to climate beliefs. For instance, conservatives tended to be more prone to climate skepticism than liberals. Recently, Hornsey et al. (2018) found that the ideological divide in climate change beliefs is more prevalent in the US, suggesting the issue of climate change has been more politicized there than in other nations. Considering that our sample was mostly European, this goes contrary to Hornsey’s findings and seems to be an international phenomenon. However, we had only a single item for Political beliefs. Further studies may attempt to replicate these findings with a more extensive instrument of political beliefs and behaviors, particularly those associated with conservatism.

Just World Beliefs correlated more strongly with CC factors than any other worldview measures. This suggests that perceptions of unpredictability and unfairness may be particularly pronounced in the case of climate change messages and that these belief violations may inspire denialist convictions. Previous findings support the notion that emphasizing the threat of climate change exacerbates climate denialism (Feinberg and Willer, 2011). However, this worldview is hardly mentioned in the climate change literature. Yet several reports seem to suggest that climate skepticism may be a coping strategy employed by people confronted with uncomfortable truths about the devastating effects of climate change. Indeed, avoiding or refusing to assimilate negative information is such a pervasive coping mechanism that it has been termed the ‘ostrich effect’ (Sullivan et al., 2010; Grzesiak-Feldman, 2013). Many scholars have noted that framing climate message in ways that align with people’s belief systems may avoid negative impacts such as climate denial (Panno et al., 2019; Goldberg et al., 2020). As we did not test this experimentally, further studies may consider testing how variations in the way climate information is presented might interact with worldviews such as BJW to determine climate beliefs, and how these in turn may impact climate action.

An interesting finding from our data related to the dark side personality measures. DSM explained about 2% of variance in climate skepticism measures, which is a typical finding in personality research. This was the only factor that remained significant after controlling for demographic variables and political views. As other measures tended to intercorrelate, personality showed a unique contribution to climate change skepticism. Importantly, measures such as Disinhibition predicted responses to impact-related factors, and did not predict factors concerning trend and attribution skepticism. Whereas traits such as Antagonism were strongly correlated with conspiracy-related factors. This suggests that certain types of skepticism are diversly impacted by dark side personality types. Somewhat parallel to current findings, previous research has found dark side traits such as disinhibition and psychopathy to predict low endorsement of health-related behaviors and intent to knowingly expose others to risk during the COVID-19 pandemic. A similar argument could be made for the association between a reckless disregard of the risk of climate change (in the form of climate change conspiracy/impact beliefs and unsustainable behaviors) and dark side personality measures. This warrants further investigation.

Given the widespread agreement among the majority of participants on human-caused climate change, it becomes evident that policy efforts should focus on reinforcing this consensus and translating it into action. Policy recommendations could include the development of educational campaigns that address the identified gap between climate change acknowledgment and the misconceptions surrounding it. Such campaigns should be tailored to the nuanced spectrum of beliefs, particularly targeting groups with Just World Beliefs, and should aim to mitigate the ‘ostrich effect’ by presenting information in a manner that resonates with their worldviews. Furthermore, the association between dark side personality traits and climate change skepticism suggests that policy-making should consider psychological factors when designing communication strategies. For example, policies could support the creation of narrative-based messaging that effectively counters conspiracy theories and disinhibited views on the impacts of climate change (Constantino and Weber, 2021). By recognizing the emotional and psychological components of climate change skepticism, policies can be more effective in promoting sustainable behaviors.

Like all studies this had limitations. It would be desirable to have other studies using our questionnaire to replicate the factor structure. We acknowledge that many of the items were taken from Wikipedia and other studies and it is possible that we missed out on some important themes which only future studies could correct. Moreover, those who work on attitude measurement may be concerned with some items being two long or too technical in use of jargon though we did pilot it. Clearly the measure, still unique in its perspective, requires more work. Next, we were restricted to self-report measures with common method variance problems. It would also have been interesting to know more about the participants’ political beliefs and attitudes given how important they appear to be on this issue. It would be most desirable to replicate the findings on a bigger and more representative population.

Whether a government or society takes steps to mitigate the threats associated with climate change is highly dependent on public beliefs. These latter in turn serve as an indicators of the likelihood of endorsement for various policies to curb carbon emissions. As such, belief in human causality is a pre-condition to acknowledge the problem of climate change in the first place. Yet discrepancies in belief systems are only part of the story when it comes to engaging different audience segments with solutions.

As with all correlational data, the current findings are merely suggestive of interesting constructs that may account for some of the observed variability in climate beliefs and do not offer insights into causal relations. However, many studies examining the impact of information on climate beliefs tend to conclude that the communication of facts and figures is insufficient in changing minds (Moser, 2010; Goldberg et al., 2020; Brosch and Steg, 2021). Thus, recognizing the dimensions and causes of climate skepticism and the characteristics associated with those that espouse such beliefs may allow communicators to move away from a knowledge deficit model (i.e., providing more information based on the premise that the public is ignorant of the facts) toward a more inclusive framework that may advance targeted communications with a higher chance of success.

The pattern of results observed here is a further indicator that there is a high level of awareness of climate change and its causes. Future research should aim to expand upon the current study’s findings by employing a multifaceted approach to understanding climate change beliefs. This could involve longitudinal studies to assess how climate change beliefs evolve over time and in response to major environmental events. Additionally, experimental research that tests different methods of presenting climate change information could be invaluable in understanding how to counteract denialist attitudes. To enhance the public’s perception of climate-related phenomena, future studies could explore the use of personalized information and storytelling that align with individuals’ existing belief systems (Hornsey and Fielding, 2017). This approach could potentially reduce defensive responses and increase the openness to scientific evidence.

Future studies would do well to assess a number of other participant variables like their endorsement of conspiracy theories, the participation in local and national politics as well as their personal behaviors associated with energy use and conservation. There has been a great deal of work on how to best address and combat conspiracy theories in general (Lewandowsky et al., 2022; O’Mahony et al., 2023). By addressing these elements, the research community can play a pivotal role in aiding society’s transition toward a more informed and proactive stance on climate change. This measure may prove useful in attempting to try to change beliefs about CC.

## Data availability statement

The original contributions presented in the study are included in the article/Supplementary material, further inquiries can be directed to the corresponding author.

## Ethics statement

The studies involving humans were approved by Norwegian Business School Ethics committee EP/2018/007. The studies were conducted in accordance with the local legislation and institutional requirements. The ethics committee/institutional review board waived the requirement of written informed consent for participation from the participants or the participants’ legal guardians/next of kin because data collected online anonymously.

## Author contributions

JL: Formal analysis, Supervision, Writing – original draft, Writing – review & editing. AF: Conceptualization, Supervision, Writing – review & editing.
